# Editorial: Hallmark of cancer: Evasion of growth suppressors

**DOI:** 10.3389/fonc.2023.1170115

**Published:** 2023-03-13

**Authors:** Christina H. Scheel, Reinhold Schäfer

**Affiliations:** ^1^ Skin Cancer Center, Department of Dermatology, Ruhr-University Bochum, Bochum, Germany; ^2^ Charité Comprehensive Cancer Center, Universitaetsmedizin Berlin, Berlin, Germany

**Keywords:** cell death, maspin, post-translational protein modification, cancer metabolism, HADHB gene, INPP4B gene

## Of TP53 and other tumor suppressors

The Frontiers collection of articles on the Hallmarks of Cancer topic “Evasion of growth suppression” includes two original papers and three review articles with the impressive number of 413 citations. Two of the three reviews focus on the tumor suppressor p53, known as the “guardian of the genome” and the most frequently mutated and/or deleted gene in human cancer (1). The article by Su et al. discusses the functional links between p53 and different types of cell death. While the function of wild-type p53 as a stress sensor for apoptotic cell death triggered by DNA damage is well known, the roles of p53 mutants as modulators of processes involved in the elimination of damaged cells is less well understood. The authors discuss the contributions of p53 mutants in autophagy-mediated cell death, in ferroptosis, characterized by iron-dependent accumulation of oxidatively damaged phospholipids, in pyroptosis, a lytic type of cell death triggered by infection, in NETosis, characterized by the release of granule components into the cytosol and chromatin decondensation associated with histone modification, as well as in cuprotosis, initiated by excessive copper concentrations. Importantly, these death pathways do not operate in isolation, and p53 mutants may provide functional links between them. The authors speculate that the complexity of molecular interactions involving p53 and its targets provide opportunities for pharmacological interventions.

In their review article, Tang et al. summarize the multiple functions of the tumor suppressor maspin, with respect to inflammation, invasion, migration, angiogenesis and immune surveillance of malignant cells Click or tap here to enter text.. Maspin is encoded by the SERPINB5 gene and a non-inhibitory member of the family of serpin protease inhibitors. Whether its function fits that of a classical tumor suppressor has been debated in the past (2). Tang et al. discuss the suppressive function of maspin as a downstream effector of p53. Thus, the authors refer to reports showing that p53 transcriptionally stimulates maspin expression. In this context, it is important to note that the transcriptional activity of p53 can be enhanced by acetylation through inhibition of histone deacetylase 1 (HDAC1) (3). Indeed, maspin has been shown to influence the acetylation levels of transcription factors and other proteins by inhibition of HDAC1. The authors emphasize that upon cellular stress and due to the functional link to p53, maspin mediates a tumor-preventive activity, which they call “self-propelling”. As an effect of this mechanism, epithelial homeostasis can be maintained. Moreover, Tang et al. speculate that the reestablishment of maspin’s epigenetic function could enhance the efficacy of chemotherapy in tumor treatment. It would be interesting to see if and how different types of mutant p53 proteins participate in such an epigenetic regulatory mechanism.

Liu et al. contribute to the topic of tumor suppressor function by adding an emerging, and increasingly recognized critical aspect by discussing effects of altered metabolism in cancer cells on the function of tumor suppressor proteins and oncoproteins. While gain-of-function mutations in oncogenes as well as the loss of tumor suppressor gene represent a root cause for altered metabolism in cancer cells, several lines of evidence suggest that metabolites in turn trigger protein modifications that modulate oncogenic pathways. Therefore, the authors discuss the possible effects of post-translational protein modifications achieved by lactylation, lipidation, S-palmitoylation, myristoylation, acetylation, succinylation and gycosylation. Thus, the article provides a list of common and less common oncoproteins and tumor suppressors that are functionally impacted by epigenetic or post-translational modification. For example, acetylation, succinylation and palmitoylation enhance nuclear p53 localization, increase protein half-life or promote transcriptional activity, resulting in enhancement of pro-apoptotic effects. Importantly, this crosstalk between the activation state of oncogenes and tumor suppressors and metabolism-induced protein modification affects some of the most prevalent oncogenes such as *KRAS, EGFR* and *MYC* and, apart from *TP53*, tumor suppressors such as *RB1* and *PTEN.*

Next, in their original research article, Li et al. discuss the role of the hydroxyacyl-CoA dehydrogenase trifunctional multi-enzyme complex subunit beta gene (*HADHB*) as a tumour suppressor in gastric adenocarcinoma. Bioinformatic analysis of expression data revealed reduced activity in this tumor type as well as in corresponding cell lines. This pattern of reduced expression was validated by independent biochemical methods and immuno-histochemistry. To show tumor-suppressive function, the authors performed functional assays based on ectopic HADHB expression and knockdown. The elucidation of HADHB-mediated phenotypic read-outs was based on assays monitoring cellular viability, proliferation, colony formation, migration, invasion, wound healing and tumorigenicity in xenotransplants. HADHB overexpression favoured these effects, while HADHB knockdown inhibited them. The authors also identified Kruppel-like factor 4 (KLF4), an evolutionarily conserved zinc finger-containing transcription factor, as a regulator of HADHB. Moreover they provided evidence for a relationship between HADHB activity and the YAP signalling pathway. Thus, pathway enrichment analysis and functional assays indicated that HADHB regulates YAP signalling, one of the main effectors of the Hippo tumor suppressor pathway.

The final article in this series focuses on the *INPP4B* gene, which encodes the inositol polyphosphate 4-phosphatase type II, a dual- specificity phosphatase. This enzyme removes a phosphate group at position 4 of the inositol ring from inositol 3,4-bisphosphate and phosphate groups from phosphotyrosine, although there is controversy regarding its function in tumorigenesis and metastasis (4). Due to PtdIns (3, 4) P2 and PIP3’s ability to recruit AKT to the plasma membrane, INPP4B is predicted to act as a tumor suppressor by inhibiting AKT recruitment, activation, and downstream PI3K signaling. Sun et al. provide evidence for the downregulation of INPP4B in low- and high-grade gliomas in an orthotopic brain glioma model in the mouse as well as in glioma cell lines. Using functional assays, the authors demonstrate that INPP4B overexpression reduced proliferation, migration and cell survival. Interestingly, modulation of INPP4B expression resulted in a reduction of PD-L1 expression and restrained T cell suppression by glioma cells. These phenotypic changes were related to downregulation of PI3K/AKT signalling. Together, these results support the notion that INPPB4 may not only inhibit the progression of the tumor but also suppress immune escape.

## Author contributions

All authors listed have made a substantial, direct, and intellectual contribution to the work and approved it for publication.
